# Gaps in treatment of epileptic seizures in a Zambian rural area

**DOI:** 10.1007/s10072-024-07456-1

**Published:** 2024-03-28

**Authors:** Richard Mambo, Andrew M. Phiri, Chiara Trevisan, Gideon Zulu, Chishimba M. Mubanga, Chembensofu Mwelwa, Isaac K. Phiri, Veronika Schmidt, Pascal Magnussen, Pierre Dorny, Sarah Gabriël, Andrea S. Winkler, Kabemba E. Mwape

**Affiliations:** 1 Department of Clinical Studies, School of Veterinary Medicine, University of Zambia, P.O BOX 32379, Lusaka, Zambia; 2 Sinda District Health Office, Ministry of Health, Chipata, Zambia; 3 Department of Public Health, Institute of Tropical Medicine, Antwerp, Belgium; 4 Department of Translational Physiology, Infectiology, and Public Health, Faculty of Veterinary Medicine, Ghent University, Merelbeke, Belgium; 5 Department of Neurology, Center for Global Health, Technical University of Munich, Munich, Germany; 6 Department of Community Medicine and Global Health, Institute of Health and Society, University of Oslo, Oslo, Norway; 7 Department of Immunology and Microbiology, Faculty of Health and Medical Sciences, University of Copenhagen, Copenhagen, Denmark; 8 Department of Biomedical Sciences, Institute of Tropical Medicine, Antwerp, Belgium; 9 Ministry of Health, Lusaka, Zambia

**Keywords:** People with epileptic seizures, Treatment gap, Neurocysticercosis, Epilepsy, Anti-seizure medications, Adherence

## Abstract

**Background:**

Epilepsy is a multifactorial neurological disorder, including parasitic infections of the brain such as neurocysticercosis (NCC). People with epileptic seizures (PWES) in low and middle-income countries often do not receive appropriate treatment, which besides epileptic seizures, may also lead to reduced quality of life and possibly death. The objective of this study was to describe gaps in treatment of epileptic seizures in a Zambian rural area.

**Methods:**

A cross-sectional study was conducted in Sinda district of Zambia between August and October 2018. PWES identified from clinic records and with the help of community healthcare workers were recruited. Two questionnaires, one to PWES and the other to local healthcare workers, were administered to describe the treatment gap.

**Results:**

A total of 146 PWES and 43 healthcare workers were interviewed. Of the 146 PWES, 131 had taken anti-seizure medication (ASM) at some point since their seizure onset, of which 49.6% were on current treatment. Only 18.3% were on continuous ASM, an overall treatment gap of 83.6%. Over 55% of healthcare workers did not know the relationship between epilepsy and NCC. The risk factors associated with lack of appropriate treatment were stock-outs of ASMs, lack of diagnostic equipment, poor patient follow-up, and PWES opting for traditional medicine.

**Conclusion:**

The treatment gap is substantial in Sinda district. The causes are multifactorial, involving shortcomings at the level of healthcare facilities, communities, and individuals. Directed training of healthcare workers and significant improvements in the supply and dispensing of ASMs will be key in substantially reducing the gap.

## Introduction

Epilepsy is a common and severe neurological disorder with the fifth-highest burden of disease following stroke, migraine, meningitis, and dementia [1]. According to WHO Inter-sectoral Global Action Plan (IGAP) on Epilepsy and Other Neurological Disorders 2022–2031, epilepsy accounted for 4.9% of the neurological disability-adjusted life years (DALY) globally in 2016 [2]. It is estimated that about 80% of people suffering from epilepsy around the globe are living in low and middle-income countries (LMICs) [3]. In sub-Saharan Africa, the epilepsy prevalence rate is 15 cases per 1000 persons [4, 5]. The Zambian epilepsy prevalence is comparable at 14.5 cases per 1000 persons [6]. The etiology of epilepsy is multifactorial. Some of the known causes include traumatic brain injury, cerebrovascular diseases, neurodegenerative diseases, and neuro-infections such as neurocysticercosis (NCC) caused by *Taenia solium* [7]. An association has been established between epileptic seizures and NCC in many low and middle-income countries [8–10]. A study conducted in Katete district of Zambia found that 57% of people with epileptic seizures (PWES) had NCC [9].

The management of epilepsy is a challenge in many LMICs [11]. The World Health Organization (WHO) considers epilepsy as one of the most cost-effective chronic conditions to treat [12]. However, the epilepsy treatment gap has been estimated to be above 90% in many resource-limited countries [6, 13, 14]. The gap is defined as the percentage of PWES who are not appropriately treated, either because of lack of access to treatment or being on inadequate treatment in a given population of people with seizures at a given time [15]. This gap has been reported in Gambia and other developing countries to be due to lack of appropriate anti-seizure medications (ASMs), especially those of newer generation, long distances to healthcare facilities, and lack of knowledge on the consequences of non-adherence to ASMs [15–17]. Lack of diagnostic equipment such as computed tomography (CT), electroencephalogram (EEG), magnetic resonance imaging (MRI), and inadequately trained healthcare workers (HCWs) also add to the poor management of PWES [18].

The commonly found ASMs in LMICs are phenobarbitone (PB), carbamazepine (CBZ), and phenytoin which are very old first-generation drugs with many side effects [18, 19]. Even when ASMs are available, many PWES prefer using superstitious forces and traditional treatments for cure, and lack information about consequences of non-adherence to treatment, hence remain untreated. This was observed in a study of 1450 people with epilepsy followed up in an urban clinic in northeast India, in which 43% discontinued treatment within 1 year [19]. PWES stop taking ASMs due to side effects, and community barriers such as stigma hinder access to treatment [15, 19]. Untreated PWES face potentially devastating social consequences and poor health outcomes, such as premature mortality, and represent a major public health problem [20, 21]. Furthermore, stigma related to epilepsy leads to low levels of education and loss of employment [6, 20].

Information on the epilepsy treatment gap is lacking in the Eastern Province of Zambia, an area highly endemic for *T. solium*, and where NCC, which is considered a major cause of seizures, is highly prevalent [9]. Therefore, this study aimed to assess the currently available treatment options for PWES, the extent of the treatment gap, and associated risk factors in a rural area.

## Materials and methods

### Study design

A cross-sectional study was conducted between August and October 2018 in the Mtandaza area in Sinda district of the Eastern Province of Zambia. The target population was all PWES in the catchment area of Mtandaza Rural Health Centre (RHC) with a history of two or more unprovoked seizures during the last 5 years. Participants were identified from RHC records and by consulting community healthcare workers (CHWs) if PWES were not registered at the RHC. All PWES above the age of 10 years were purposively recruited as the number in the catchment was manageable. A proportion of HCWs who attended to patients at each of the 23 healthcare facilities of Sinda district were recruited for the questionnaire survey.

### Study area

Mtandaza RHC is located south-east of Sinda district and has a catchment population of 16,127 (2016 clinic headcount records). The most common ethnic group of the Mtandaza area is the Chewa speaking people. They practice subsistence agriculture, raising animals and growing crops. People’s homes in this area are of adobe and have few sanitary facilities, which in many instances are not used allowing free-roaming pigs to access human feces [22]. The area was chosen because of available data for PWES through clinic records and the endemicity of human and porcine cysticercosis [23, 24].

### Study participants

Participants were PWES ≥ 10 years of age, willing to participate in all aspects of the study, and able to sign the written informed consent. PWES who were not able to read and write were asked to print their right thumb on the informed consent form after reading and explaining to them the information sheet in their local language. Assent was sought from minors, with a parent or a guardian consenting on their behalf. Self-reported pregnant women and those who were seriously ill were excluded from the study as well as PWES < 10 years of age.

HCWs responsible for the management of seizures in Sinda district were separately recruited from 14 health centers and nine health posts. The targeted HCWs were the enrolled nurses, registered nurses, nurse midwives, clinical officers, and medical doctors. Enrolled nurses/midwives study nursing for only 2 years and obtain a certificate, while clinical officers and registered nurses/midwives obtain a diploma after completion of a 3-year course. One HCW from each health post and at least two HCWs from each health center, depending on the number of professional HCWs available at the facility were included for the questionnaire interview. The experience of a HCW was not taken into account when choosing who the questionnaire should be administered to.

### Questionnaire surveys

A questionnaire adapted from Mwape et al*.* (2015) [9] was administered to all eligible PWES and another questionnaire to HCWs responsible for the management of PWES in Sinda district (complete questionnaires are available in supplementary materials). Both questionnaires were administered by the researcher.

#### Questionnaire for people with epileptic seizures

PWES were asked about age, sex, education level, seizure status, and whether they were on ASMs, and if not on ASMs, PWES were asked to give reasons. Treatment adherence was investigated only in PWES who reported to be on ASMs. Adherence is the extent to which patients take medications as prescribed by their healthcare providers [18]. Data was collected on the type of ASMs, dosage and frequency of treatment, reasons for not taking ASMs regularly or as prescribed, alcohol abuse, distance to the clinic, barriers to drug collection, and if there were any side effects that arose from ASMs. For PWES on ASMs, the consistency of ASM intake from the date of drug collection until the day of the questionnaire interview was checked by counting the tablets, and it was used as a proxy measure of adherence to ASM treatment. After the interview, the importance of adherence to treatment was emphasized.

#### Questionnaire for healthcare workers

A questionnaire was used to assess HCWs’ practices towards epilepsy and their knowledge on the relationship between epilepsy and NCC. Data was also collected on professional qualifications, years of experience, approaches to epilepsy diagnosis, epilepsy prevalence estimates and number of PWES seen per week at their clinics, challenges faced when attending to PWES, titration of ASMs, and knowledge on the management of epileptic seizures. Health education on the relationship between NCC and epilepsy, as well as on prevention and control of NCC, was given to the HCW/s after the interview. Additionally, in the framework of other ongoing projects, trainings were organized for HCWs in the area of epilepsy management.

### Observation of healthcare facility service

The healthcare service was assessed based on the accessibility of the healthcare facilities by the population, the degree to which HCWs adhered to the epilepsy management outlined in the Zambia standard treatment guidelines [25], availability of nurses, clinical officers, and medical doctors. Checks of pharmacy/treatment rooms on availability and types of ASMs and diagnostics were done after the questionnaire survey. In addition, the 2016/2017 training curriculum for clinical officers used in learning institutions of Zambia was reviewed to see what it covered concerning epilepsy and NCC.

### Data management and statistical analysis

The questionnaires were set up electronically, and data was collected using Epicollect5 (https://five.epicollect.net). Data collected was downloaded as comma-separated values (CSV) file and was directly exported in R statistical software for descriptive data analysis using R version 4.0.2 [26]. All participant sensitive data were pseudonymized and handled confidentially following good research practices.

## Results

### Study participants

#### People with seizures

**Fig. 1:**
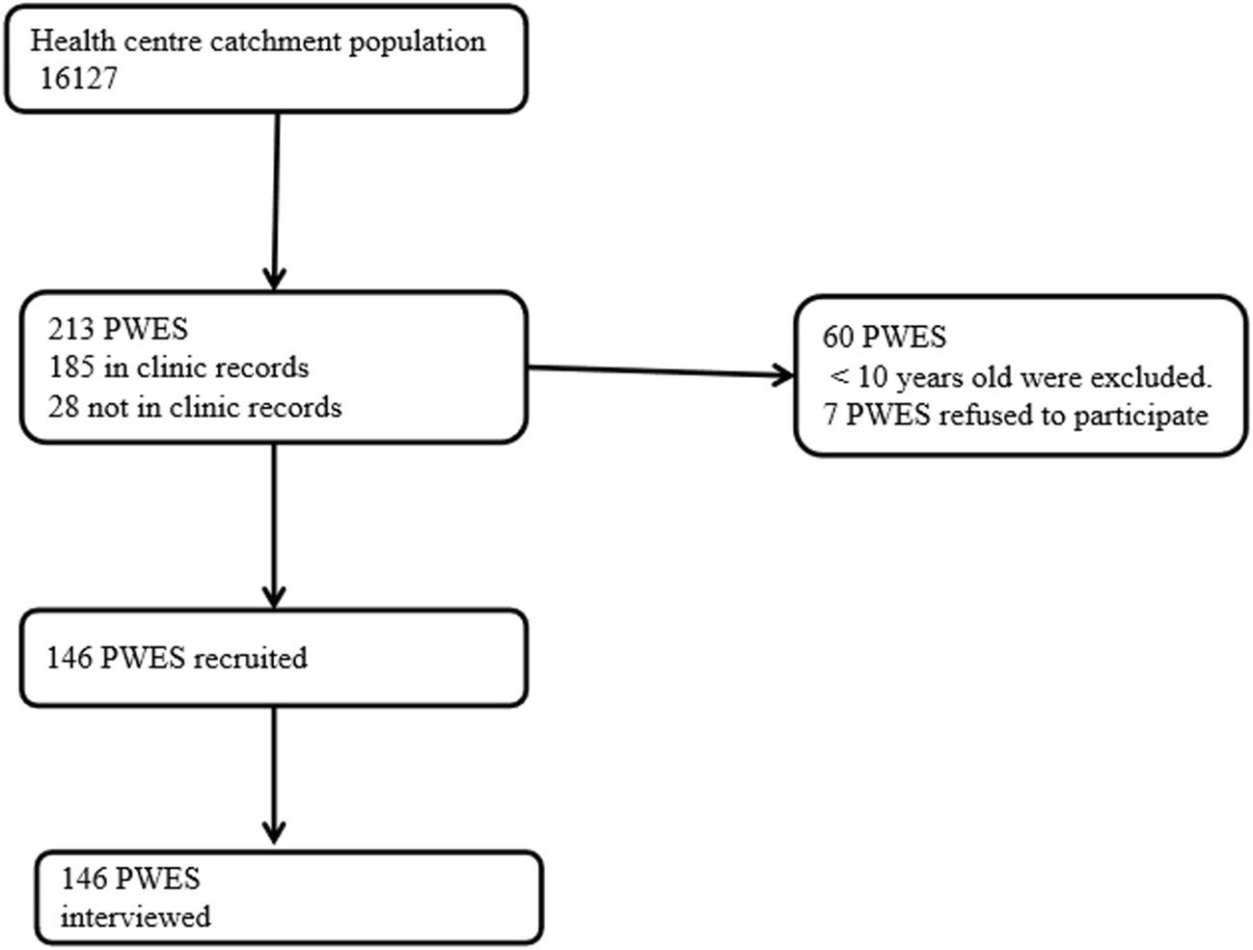
Schematic presentation of the participants included in the study

Figure 1 shows the flow for PWES enrolled into the study. Our approach identified 213 PWES in the catchment area. Of these, 185 were through the RHC records, and 28 were through consulting CHWs. The 28 PWES never attended a clinic for either diagnosis or seizure treatment. Forty-four PWES reported in the RHC records, and 16 reported after consulting the CHWs were < 10 years old and were thus excluded from the study. Seven refused to participate in the study. Therefore, a total of 146 participants were recruited for the study. Among the participants, 67 (45.9%) were female, and one third (44) were < 20 years old. The median age of the study participants was 31 years.

### Questionnaire results for PWES

#### Epilepsy diagnosis

Of the 146 PWES interviewed, 17 (11.6%) were diagnosed with epilepsy by a medical doctor at a hospital, 42 (28.8%) at the local clinic, and 75 (51.4%) were identified in their communities by elders who then reported to the CHWs after noticing seizures. These were later diagnosed by either a clinical officer or a nurse at their local healthcare facility when they went to seek treatment. Twelve PWES (8.2%) had never been to the clinic for either diagnosis or management of seizures and were identified via the CHWs after noticing recurrent seizures.

**Fig. 2:**
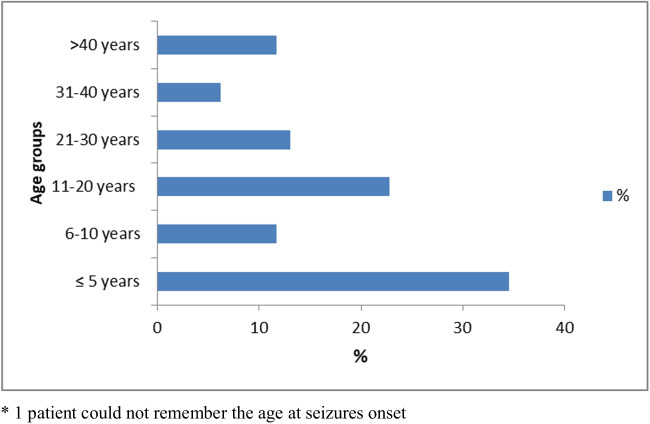
Reported patients’ age at seizure onset. One patient could not remember the age of seizure onset

Fifty (34.5%) PWES had their first seizure at or before the age of 5 years (Fig. 2). Eighty-six (58.9%) PWES had their last seizures within the month of the interviews; 34 (23.3%) between 1 and 6 months before the interview, and 26 (17.8%) reported to have had their last seizure more than a year before the interview**.**

#### Questionnaire for healthcare workers

Forty-three HCWs from 23 health facilities of Sinda district were interviewed. Of those, 58% were males, 14 were enrolled nurses, 11 registered nurses, 11 clinical officers, four nurse midwives, two medical doctors, and one mental health nurse. Table 1 shows the years of professional experience of the HCWs.

**Table 1:** Healthcare worker’s years of professional experience

Experience	*n*	%
< 1 year	13	30.2
1–4 years	21	48.8
5–9 years	4	9.3
≥ 10 years	5	11.6
Total	43	100

The HCWs expressed sufficient knowledge of the epilepsy treatment guidelines; however, 24 (55.8%) of them did not know the relationship between epilepsy and NCC. The HCWs estimated the prevalence of active seizures in their health facilities to be on average between 6 and 11 cases per 1000 persons.

### Epilepsy treatment gaps

Of the 146 PWES interviewed, 131 (89.7%) reported to have taken ASMs at some point since the onset of their seizures, while 15 (10.3%) had never been on any treatment. Out of the 131 PWES, 65 (49.6%) had reported to be currently on ASMs. Of these, only 24 (*N* = 131, 18.3%) were on continuous ASM resulting in an overall treatment gap of 83.6% (*N* = 146), while 41 (31.3%) had been without drugs for at least a month during the previous year.

**Fig. 3:**
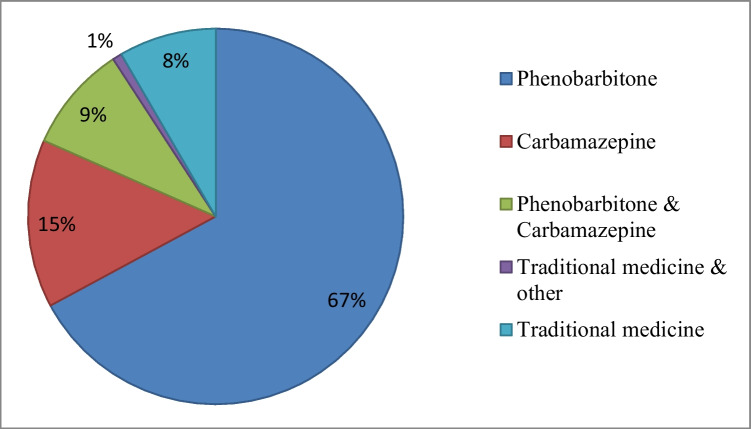
Reported treatment options for the 146 people with seizures in Sinda district in 2018

Sixty-six (50.4%) of the 131 PWES sought ASM treatment only when having an epileptic seizure among which 11 (8.4%) were exclusively on traditional medicine at the time of the interview and one (0.8%) was on traditional medicine and other unknown medicine. Twelve (9.2%) respondents reported taking more than one ASM interchangeably. The available ASMs in the study area were PB (88, 67.2%) and CBZ (19, 14.5%) as shown in Fig. 3. In the study area, traditional medicine used included plant, animal, and mineral-based medicines, and spiritual therapies applied singularly or in combination to treat illnesses. There was no special management of PWES with NCC in the study area apart from the use of ASMs.

### Factors contributing to the epileptic seizures treatment gap

**Fig. 4:**
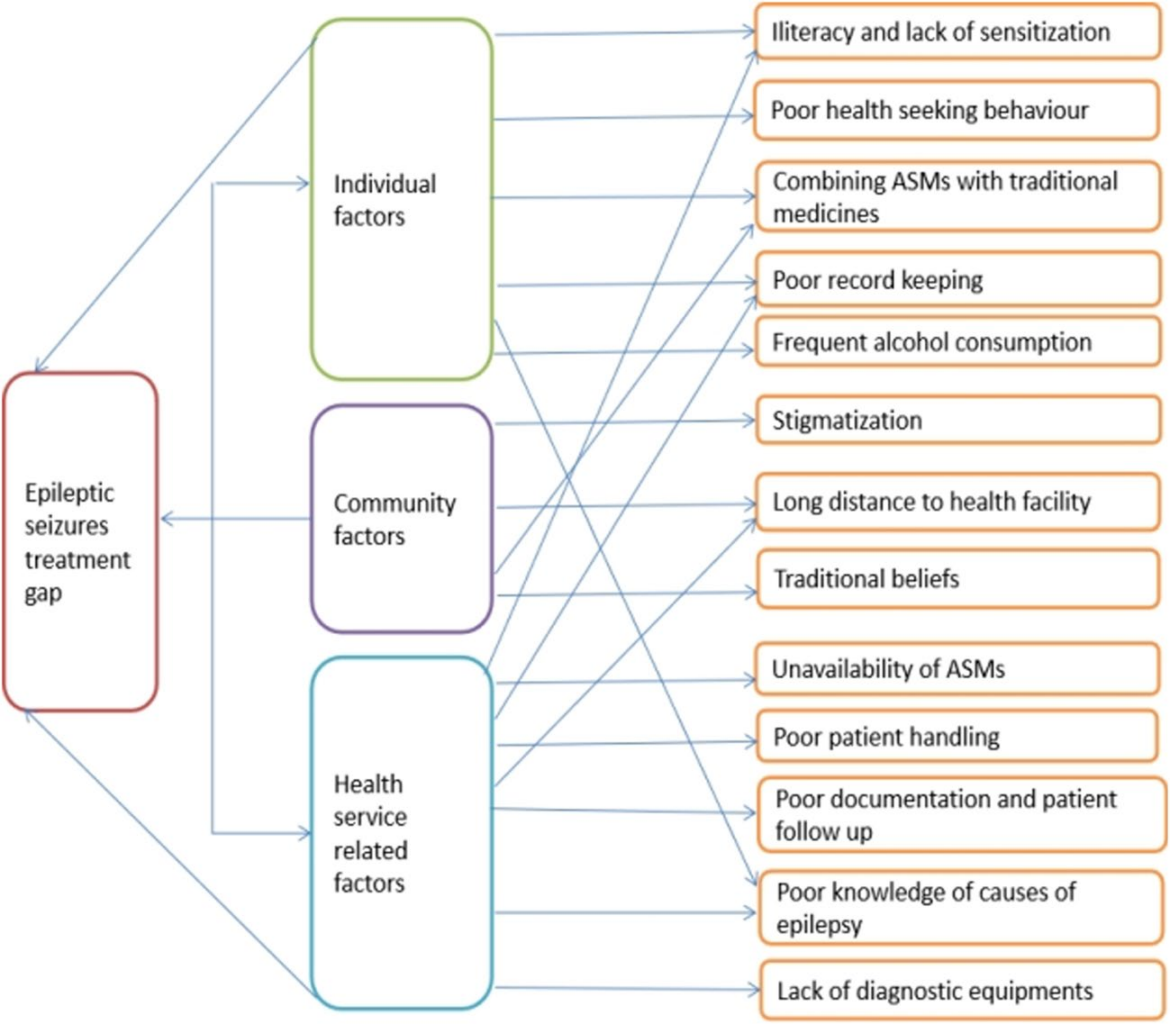
Schematic presentation of factors leading to high treatment gaps in the study area

The factors that contributed to the high treatment gap were grouped into three categories; individual, community, and healthcare service-related. Some of these factors were crosscutting as shown in Fig. 4.

#### Individual factors

These included illiteracy, poor health-seeking behavior, poor keeping of treatment records (such as appointment and treatment cards), combining ASMs with traditional medicine, and excessive alcohol consumption. More than half (74, 50.7%) of PWES had never been to school; 64 (43.8%) attended at least some early grades of primary education, and eight (5.5%) reached high school grades, with only one of all PWES completing secondary education. PWES reported dropping out of school due to seizures, lack of finances, participating in cattle herding, and cultural practices such as traditional dances (*gule wamukulu*).

Among the 131 PWES that at some point in time took ASMs, 105 (*N* = 131, 80.2%) reported to have been without ASMs for over a period of one month. According to 44 PWES, (N = 105, 41.9%) non-availability of ASMs in health facilities was the primary cause for non-adherence to ASMs. Among the 65 PWES on current medication, 41 (*N* = 65, 63.1%) reported having lived for more than 1 month without ASMs during the last 1 year. This was attributed to the non-availability of drugs at the local clinic by 32 PWES (*N* = 41, 78.0%), while nine (*N* = 41, 22.0%) had the following reasons: stopped experiencing seizures, had the belief that they were cured, had no one to help them with drug collection when they got sick, and ASMs were considered not effective, and thus, they opted for traditional medicine. Furthermore, among the nine PWES, three reported that they had lost treatment records, which were needed to access healthcare services. Three (2.3%) PWES on treatment reported that they drank alcohol almost every day to forget the problems that come with their seizures.

#### Community factors

Community factors consisted of stigmatization, long distances to health facilities, and traditional beliefs. Four parents reported collecting ASMs for their teenage children who feared being stigmatized if they went to the clinic. Furthermore, 72 PWES (*N* = 131, 55.0%) that had used ASMs lived more than 5 km from the clinic where they collected drugs. Of these, 38 (*N* = 131, 29.0%) walked to go for drug collection, with 33 (*N* = 131, 25.2%) having access to bicycles, and one (*N* = 131, 0.8%) who was always taken to the clinic on an oxcart.

Fifteen (*N* = 146, 10.3%) of the total PWES interviewed said epilepsy was considered by the community to be a disease that was due to witchcraft or a spiritual sickness and came as punishment for indiscipline, and they believed that only traditional medicine could cure it.

#### Healthcare service-related factors

The factors included the unavailability of ASMs, poor patient handling, poor documentation and patient follow-up, poor knowledge of NCC, inadequate or no diagnostic equipment, and catchment areas too large to service adequately. Non-availability of ASMs was a primary contributing factor to non-adherence to treatment. Thirty-eight (*n* = 43, 88.4%) HCWs reported erratic availability of ASMs, stating that three to four times in a year a healthcare facility would have no ASMS for more than 1 month.

HCWs reported challenges with the diagnosis for both epilepsy and NCC due to a lack of equipment and logistics. Out of the 43 HCWs interviewed, 24 (55.8%) did not know the relationship between epilepsy and NCC out of which 18 (41.9%) said they had never heard of NCC.

Twenty-nine (67.4%) HCWs reported facing challenges of follow-up with PWES, such as patients not coming with treatment records to show which ASM they were taking and missing scheduled appointments, and other PWES easily forgot instructions on taking ASMs. HCWs could not give an affirmative response to whether they tapered or increased ASM dosage to determine the right dose for new patients.

Four (9.3%) HCWs reported that some PWES had developed a preference for a specific ASM and did not accept to be given alternative ASMs. If their ASM of choice was out of stock, they would rather stay without any drug until their drug of choice was available. The reasons given to HCWs by PWES for such preferences were that some ASMs caused dizziness while others said a particular ASM did not suppress seizures in them. Further, of the 131 who had taken treatment at some point, 40 (30.5%) reported side effects such as headache (16), dizziness (11), vision problems (2), amnesia (2), and reported body weakness, abdominal pains, and vomiting (11).

When the 131 PWES were asked how they perceived the care given at the clinic when they went for drug refills, 107 (81.7%) said they were attended to well, eight (6.9%) perceived being given poor care by HCWs, six (4.6%) felt neglected, and 10 (7.6%) said they did not know. Twenty-two PWES (16.8%) reported that they faced other challenges when they went to the clinic for drug supplementation such as long waiting times as they had to queue up with other patients. This at times resulted in having a seizure while waiting. Other challenges were drug stock-outs which resulted in added costs when referred to a faraway alternative facility for drug collection and parents or guardians not being allowed to collect drugs for them. It was challenging when frequent trips to the clinic were needed due to inadequate quantities of drugs supplied.

### Observation at healthcare facilities

Observations were conducted in 23 health facilities in Sinda district. Every health facility had at least one nurse. Nine health centers had a clinical officer and the hospital had two medical doctors. Nineteen of the health facilities had the 2016 Zambian Ministry of Health standard treatment guidelines available. These guidelines describe the management of epileptic seizures and treatment of taeniosis but not cysticercosis or NCC. Furthermore, the 2016/2017 training curriculum for clinical officers did not cover cysticercosis and NCC management. Most health facilities stock PB and CBZ as evidenced on their stock records. At the time of the study, both PB and CBZ were out of stock in 17 out of the 23 health facilities, and six facilities had PB only. The ASM stock-out was reported to be a result of non-availability at the district pharmacy and was noticed to have lasted for more than a month in many instances. It was noted that ASMs were not part of the Zambian Ministry of Health essential drugs kit package for health centers and that they had to be ordered separately.

## Discussion

This is a recent study in Zambia to look at the epileptic seizure treatment gap in an area highly endemic for *T. solium*, a major cause of seizures. The treatment gap of 83% may be an underestimation as the study only included PWES of at least 10 years of age. The findings are comparable to those reported elsewhere in sub-Saharan Africa, where treatment gaps of more than 80% were recorded [9, 19, 27]. The lack of adequate treatment could explain the findings by Nau et al. [28] who reported cognitive impairment and reduced quality of life among people with epilepsy in the Eastern Province of Zambia.

The treatment gap was attributed to individual patient, community, and healthcare service factors. PWES could not adhere to treatment due to shortages of drugs at health facilities and lack of knowledge of the importance of taking ASMs for the prevention of seizures. Some PWES abandoned taking medication once seizures were temporarily suppressed, consequently leading to uncontrolled seizures. Other studies have reported PWES who started treatment but stopped after some time, and only resumed when experiencing seizures [19, 28]. About 30% of PWES on ASMs reported side effects of the medication during an interview. Gillham et al. [29] estimated the incidence of ASM side effects to be as high as 58%. The lower reporting rate in our study could be attributed to the patients ignoring, avoiding, or disregarding the need to inform the HCWs of the side effects of the ASMs or the resolution of these effects once the medication was discontinued. Equally, there is a poor follow-up to PWES by HCWs to monitor adherence and the efficacy of ASMs. Screening for ASM side effects may identify toxicity and guide drug changes to reduce side effects and possibly improve subjective health outcome [30]. Titration of the ASMs to determine the most appropriate dose for every patient was never carried out. This could be contributing to PWES not observing a reduction in the frequency of seizures or experiencing severe side effects thus leading to abandoning treatment [31]. Adopting the use of recently developed mobile applications can allow providers to follow up and work closely with PWES to improve their adherence and self-management skills [32].

The literacy levels of the people for Eastern Province remain quite low at 47% for women and 73% for men [33]. The low literacy levels observed in this study might have contributed to non-adherence to the prescribed treatment. This study reports that 50.7% of recruited PWES have no formal education. Low literacy levels have been reported to lead to either over-consumption or under-consumption of ASMs [34–36]. Under-dosage may lead to seizure relapses, while over-dosage of ASMs exposes patients to higher risks of ASM dose-dependent side effects [37]. About 2% of PWES interviewed admitted that they drank alcohol almost daily. Alcohol consumption has been reported to be associated with impaired adherence to medication, particularly with taking medication off schedule [35, 38]. Thus, sensitizations on the effects of alcohol on medication would be a key for enhancing adherence to treatment.

The local community’s perception of seizures also contributed to the high treatment gap. People in the area associated seizures to spiritual illness resulting from witchcraft and did not see ASM treatment as a solution, hence resorting to traditional medicine. About 10% of PWES reported that their seizures were due to witchcraft and that only traditional medicine could cure them. Epilepsy or any other seizures are believed to be an evil spell that comes as punishment for one going against cultural beliefs. Such beliefs and explanations about seizures have been reported to play a major role in the treatment gap as they influence health-seeking behavior [3, 16]. Negative beliefs and attitudes about seizures contribute to non-adherence to ASMs and promote stigma [17]. The stigma associated with epileptic seizures contributes significantly to the psychological and social burden of PWES. Many PWES delay accessing treatment for fear of stigmatization hence the condition worsening to the extent of causing irreparable damage [20, 21]. Modification of communities’ beliefs and attitudes, through dialog and information sharing, would help to bring epilepsy “out of the shadows,” rule out misconceptions, reduce stigma, improve treatment seeking and adherence, and eventually narrow the treatment gap [16, 17, 19]. Furthermore, the government through Ministry of Health should strive to create better opportunities for knowledge sharing for PWES, their relatives, and the community. Digital interventions such as animated stories on epilepsy would help to increase knowledge uptake in the communities [32, 39].

Even when PWES visited the clinic, etiological diagnosis of the seizures was not possible, and presumptive treatment was thus given. NCC has been reported as a leading cause of seizures in the study area [9], yet more than 50% of HCWs did not know the relationship between epilepsy and NCC seizures. The high occurrence of NCC in the area requires that HCWs have adequate knowledge on its existence and how to properly manage the condition. This low knowledge among HCWs could also be the cause for inadequate preventive actions for NCC in the communities. The government should create opportunities for public–private partnerships and collaborate with WHO and IGAP on the procurement of neuroimaging equipment and capacity building of HCWs.

The recurring stock-out of ASMs in health facilities was among the reported contributors to the treatment gap. A study in Zambia on the availability of ASMs in 111 pharmacies in Lusaka and Southern provinces revealed that availability was inconsistent in all the pharmacies, and 45.9% of government pharmacies had no ASM in stock [17]. The situation could even be worse for places further away from Lusaka. This current study reports ASMs non-availability as a primary contributor to the treatment gap. About 88% of the HCWs reported that ASMs were not always available in their health facilities, and about 77% of PWES mentioned non-availability of ASMs at their nearest health facility as the reason for not adhering to treatment. This, together with long distances to health facilities, makes it unlikely that adherence to treatment will be sustained. The government should consider adding ASMs of better efficacy on the essential medicines list to mitigate the challenges of stock-outs. Furthermore, strengthening the current primary healthcare system would help reduce the treatment gap [15]. This would require integrating epilepsy into other existing chronic diseases programs such as HIV/AIDS and involving primary HCWs such as the CHWs in distributing ASMs to registered patients so that PWES would only be required to cover long distances to the clinic for diagnosis, initial prescription, and periodic assessments and when there are ASMs side effects [40]. This would be in line with the WHO-IGAP strategic objective number 5 “to strengthen the public health approach to epilepsy—constitutes a unique opportunity and clear mandate for the global epilepsy community to take concerted and multipronged action in addressing the unmet needs of people with epilepsy” [2].

The study had several limitations. Firstly, the majority of PWES in our study were defined as PWES by the community elders and later by a local nurse or clinical officer based on seizure episodes only, which could have led to a false diagnosis of epilepsy and leaving out many PWES. Secondly, due to stigma, there was a possibility that some PWES were not captured for recruitment. PWES under the age of 10 years were not recruited as well. This might have led to under-reporting of the treatment gap. Lastly, this study looked at adherence to ASMs as being found with drugs at only one point during interviews, as opposed to checking clinic visit records, pharmacy records, and monitoring of seizures through home visits.

In conclusion, this study demonstrated that gaps in the treatment of epileptic seizures exist. Individual seizure patient, community, and healthcare-related factors, singly and collectively, contributed to the high treatment gap. To reduce this gap, national budgets should prioritize appropriate ASMs and add them to the essential drugs health center kit. Further, there is a need for adequate counseling of patients on treatment adherence, improving diagnostic facilities in affected communities, and training of more specialized HCWs. Raising awareness of epilepsy and its causes among the communities and HCWs should be among the key solutions to reducing the treatment gap. NCC should be considered as a probable cause of epilepsy in the Eastern Province of Zambia, and preventive health education on *T. solium* should be intensified.

## Data Availability

The authors confirm that the data supporting the findings of this study are available within the article and its supplementary material. Raw data that support the findings of this study are available from the corresponding author, upon reasonable request.

## References

[1] Feigin VL, Abajobir AA, Abate KH, Abd-Allah F, Abdulle AM, Abera SF, Abyu GY, Ahmed MB, Aichour AN, Aichour I, Aichour MT (2017) Global, regional, and national burden of neurological disorders during 1990–2015: a systematic analysis for the Global Burden of Disease Study 2015. Lancet Neurol 16(11):877–897 28931491 10.1016/S1474-4422(17)30299-5 PMC5641502

[2] World Health Organization (2023) Intersectoral global action plan on epilepsy and other neurological disorders 2022–2031 10.1227/neu.0000000000001976 35383690

[3] Diop AG, de Boer HM, Mandlhate C, Prilipko L, Meinardi H (2003) The global campaign against epilepsy in Africa. Acta Trop 87(1):149–159 12781390 10.1016/S0001-706X(03)00038-X

[4] Preux PM, Druet-Cabanac M (2005) Epidemiology and aetiology of epilepsy in sub-Saharan Africa. Lancet Neurol 4(1):21–31 15620854 10.1016/S1474-4422(04)00963-9

[5] Winkler AS, Kerschbaumsteiner K, Stelzhammer B, Meindl M, Kaaya J, Schmutzhard E (2009) Prevalence, incidence, and clinical characteristics of epilepsy—a community-based door-to-door study in northern Tanzania. Epilepsia 50(10):2310–2313 19583783 10.1111/j.1528-1167.2009.02184.x

[6] Birbeck GL, Kalichi EM (2004) Epilepsy prevalence in rural Zambia: a door-to-door survey. Tropical Med Int Health 9(1):92–95 10.1046/j.1365-3156.2003.01149.x 14728612

[7] Annegers JF, Rocca WA, Hauser WA (1996) Causes of epilepsy: contributions of the Rochester epidemiology project. In: Mayo Clinic Proceedings, vol 71, no 6. Elsevier, p 570–575 10.4065/71.6.570 8642886

[8] Assane YA, Trevisan C, Schutte CM, Noormahomed EV, Johansen MV, Magnussen P (2017) Neurocysticercosis in a rural population with extensive pig production in Angonia district, Tete Province. Mozambique Acta Tropica 1(165):155–160 10.1016/j.actatropica.2015.10.018 PMC6333921 26519884

[9] Mwape KE, Blocher J, Wiefek J, Schmidt K, Dorny P, Praet N, Chiluba C, Schmidt H, Phiri IK, Winkler AS, Gabriël S (2015) Prevalence of neurocysticercosis in people with epilepsy in the Eastern Province of Zambia. PLoS Negl Trop Dis 9(8):e0003972 26285031 10.1371/journal.pntd.0003972 PMC4540454

[10] Carpio A, Romo ML (2014) The relationship between neurocysticercosis and epilepsy: an endless debate. Arq Neuropsiquiatr 72:383–390 24863516 10.1590/0004-282X20140024

[11] Winkler AS (2013) Epilepsy and neurocysticercosis in sub-Saharan Africa. In: Foyaca Sibat H (ed) Novel aspects on cysticercosis and neurocysticercosis. InTech, p 307–340

[12] World Health Organization. Department of Mental Health, Substance Abuse, World Health Organization. Mental Health Evidence, & Research Team (2005) Mental health atlas 2005. World Health Organization

[13] Meyer AC, Dua T, Boscardin WJ, Escarce JJ, Saxena S, Birbeck GL (2012) Critical determinants of the epilepsy treatment gap: a cross-national analysis in resource-limited settings. Epilepsia 53(12):2178–2185 23106784 10.1111/epi.12002 PMC3809906

[14] Kale R (2002) The treatment gap. Epilepsia 43:31–33 12190976 10.1046/j.1528-1157.43.s.6.13.x

[15] Coleman R, Loppy L, Walraven G (2002) The treatment gap and primary health care for people with epilepsy in rural Gambia. Bull World Health Organ 80:378–383 12077613 PMC2567785

[16] Mbuba CK, Ngugi AK, Fegan G, Ibinda F, Muchohi SN, Nyundo C (2012) Risk factors associated with the epilepsy treatment gap in Kilifi, Kenya: a cross-sectional study. Lancet Neurol 11(8):688–696 22770914 10.1016/S1474-4422(12)70155-2 PMC3404220

[17] Chomba EN, Haworth A, Mbewe E, Atadzhanov M, Ndubani P, Kansembe H (2010) The current availability of antiepileptic drugs in Zambia: implications for the ILAE/WHO “out of the shadows” campaign. Am J Trop Med Hyg 83(3):571 20810822 10.4269/ajtmh.2010.10-0100 PMC2929053

[18] Ernawati I, Wardah Rahmatul Islamiyah S (2018) How to improve clinical outcome of epileptic seizure control based on medication adherence? A literature review. Open Access Macedonian J Med Sci 6(6):1174 10.3889/oamjms.2018.235 PMC6026415 29983823

[19] Das K, Banerjee M, Mondal GP, Devi LG, Singh OP, Mukherjee BB (2007) Evaluation of socio-economic factors causing discontinuation of epilepsy treatment resulting in seizure recurrence: a study in an urban epilepsy clinic in India. Seizure 16(7):601–607 17576079 10.1016/j.seizure.2007.04.008

[20] Atadzhanov M, Haworth A, Chomba EN, Mbewe EK, Birbeck GL (2010) Epilepsy-associated stigma in Zambia: what factors predict greater felt stigma in a highly stigmatized population? Epilepsy Behav 19(3):414–418 20851056 10.1016/j.yebeh.2010.08.017 PMC3005974

[21] Ding D, Wang W, Wu J, Ma G, Dai X, Yang B, Wang T, Yuan C, Hong Z, de Boer HM, Prilipko L (2006) Premature mortality in people with epilepsy in rural China: a prospective study. Lancet Neurol 5(10):823–827 16987728 10.1016/S1474-4422(06)70528-2

[22] Thys S, Mwape KE, Lefevre P, Dorny P, Marcotty T, Phiri AM, Phiri IK, Gabriël S (2015) Why latrines are not used: communities’ perceptions and practices regarding latrines in a Taenia solium endemic rural area in Eastern Zambia. PLoS Negl Trop Dis 9(3):e0003570 25739017 10.1371/journal.pntd.0003570 PMC4352092

[23] Mwape KE, Phiri IK, Praet N, Speybroeck N, Muma JB, Dorny P, Gabriël S (2013) The incidence of human cysticercosis in a rural community of Eastern Zambia. PLoS Negl Trop Dis 7(3):e2142 23556026 10.1371/journal.pntd.0002142 PMC3605208

[24] Mwape KE, Phiri IK, Praet N, Muma JB, Zulu G, Van den Bossche P, De Deken R, Speybroeck N, Dorny P, Gabriël S (2012) *Taenia solium* Infections in a rural area of Eastern Zambia - a community based study. PLoS Negl Trop Dis 6(3):e1594 22479664 10.1371/journal.pntd.0001594 PMC3313923

[25] Ministry of Health (2017) Standard treatment guidelines. Vol: 04, Lusaka, Zambia. A book

[26] R Core Team, R (2020) R Core Team R: a language and environment for statistical computing. R Core Team, Vienna

[27] Chin JH (2012) Epilepsy treatment in sub-Saharan Africa: closing the gap. Afr Health Sci 12(2):186–192 23056026 10.4314/ahs.v12i2.17 PMC3462534

[28] Nau AL, Mwape KE, Wiefek J, Schmidt K, Abatih E, Dorny P, Praet N, Chiluba C, Schmidt H, Phiri IK, Winkler AS (2018) Cognitive impairment and quality of life of people with epilepsy and neurocysticercosis in Zambia. Epilepsy Behav 1(80):354–359 10.1016/j.yebeh.2017.10.042 29221763

[29] Gillham R, Baker G, Thompson P, Birbeck K, McGuire A, Tomlinson L, Eckersley L, Silveira C, Brown S (1996) Standardisation of a self-report questionnaire for use in evaluating cognitive, affective and behavioural side-effects of anti-epileptic drug treatments. Epilepsy Res 24(1):47–55 8800634 10.1016/0920-1211(95)00102-6

[30] Gilliam FG, Fessler AJ, Baker G, Vahle V, Carter J. Systematic screening allows reduction of adverse antiepileptic drug effects: a randomized trial. *Neurology*. 2004 62(1):23–7. 14718691 10.1212/WNL.62.1.23

[31] Fishman J, Kalilani L, Song Y, Swallow E, Wild I (2018) Antiepileptic drug titration and related health care resource use and costs. J Managed Care Special Pharm 24(9):929–38 10.18553/jmcp.2018.17337 PMC10397847 29486142

[32] Mirpuri P, Chandra PP, Samala R, Agarwal M, Doddamani R, Kaur K, Tripathi M (2021) The development and efficacy of a mobile phone application to improve medication adherence for persons with epilepsy in limited resource settings: a preliminary study. Epil Behav 116:107794 10.1016/j.yebeh.2021.107794 33578224

[33] CSO M (2013) ICF international. Zambia demographic and health survey, 2014

[34] Carpentier N, Jonas J, Frismand S, Vignal JP, Rikir E, Baumann C, Lapicque F, Saint-Marcoux F, Vespignani H, Maillard L (2013) Direct evidence of nonadherence to antiepileptic medication in refractory focal epilepsy. Epilepsia 54(1):e20–e23 23148705 10.1111/j.1528-1167.2012.03695.x

[35] Cook RL, Sereika SM, Hunt SC, Woodward WC, Erlen JA, Conigliaro J (2001) Problem drinking and medication adherence among persons with HIV infection. J Gen Intern Med 16(2):83–88 11251758 10.1111/j.1525-1497.2001.00122.x PMC1495171

[36] Hegazi A, Bailey RL, Ahadzie B, Alabi A, Peterson K (2010) Literacy, education and adherence to antiretroviral therapy in The Gambia. AIDS care 22(11):1340–1345 10.1080/09540121003693514 20711888

[37] Scott RA, Lhatoo SD, Sander JW (2001) The treatment of epilepsy in developing countries: where do we go from here? Bull World Health Organ 79:344–351 11357214 PMC2566404

[38] Hamerle M, Ghaeni L, Kowski A, Weissinger F, Holtkamp M (2018) Alcohol use and alcohol-related seizures in patients with epilepsy. Front Neurol 5(9):401 10.3389/fneur.2018.00401 PMC5996121 29922217

[39] Côté J, Beaudet L, Auger P, Rouleau G, Chicoine G, Léger V, Nguyen DK (2021) Evaluation of a web-based virtual nursing intervention to support self-management among adults with epilepsy: a mixed-methods study. Epil Behav 114:107581 10.1016/j.yebeh.2020.107581 33246896

[40] Jilek-Aall L, Rwiza HT (1992) Prognosis of epilepsy in a rural African community: a 30-year follow-up of 164 patients in an outpatient clinic in rural Tanzania. Epilepsia 33(4):645–650 1628578 10.1111/j.1528-1157.1992.tb02341.x

